# Internal Acoustic Meatus: Morphological Peculiarities and Clinical Implications

**DOI:** 10.3390/audiolres16040116

**Published:** 2026-08-10

**Authors:** Angela Babuci, Laila Ashkar, Zinovia Zorina, Svetlana Capcelea, Silvia Stratulat, Ilia Catereniuc, Lora Gitu, Doina Botnaru, Marin Buracovschi, Igor Cemortan, Nicolae Chele, Andrei Mostovei, Gabriela Motelica, Ion Dabija, Stanislav Strisca, Mihaela Dandara, Liliana Nastas, Sergiu Beliniuc, Elena Stepco, Svetlana Lozovanu, Olga Cheptanaru, Sofia Lehtman

**Affiliations:** 1 Department of Anatomy and Clinical Anatomy, Nicolae Testemitanu State University of Medicine and Pharmacy, 2004 Chisinau, Moldova; zinovia.zorina@usmf.md (Z.Z.); ilia.catereniuc@usmf.md (I.C.); doina.botnaru@usmf.md (D.B.); 2 Wolfson Medical Center, Holon 5822012, Israel; ashkarluly@gmail.com; 3 Department of Molecular Biology and Human Genetics, Nicolae Testemitanu State University of Medicine and Pharmacy, 2004 Chisinau, Moldova; svetlana.capcelea@usmf.md (S.C.); igor.cemortan@usmf.md (I.C.); 4 Department of Biochemistry and Clinical Biochemistry, Nicolae Testemitanu State University of Medicine and Pharmacy, 2004 Chisinau, Moldova; silvia.stratulat@usmf.md; 5 Department of Family Medicine, Nicolae Testemitanu State University of Medicine and Pharmacy, 2004 Chisinau, Moldova; lora.gitu@usmf.md; 6 Department of Otorhinolaryngology, Nicolae Testemitanu State University of Medicine and Pharmacy, 2004 Chisinau, Moldova; marinburacovschi@gmail.com; 7 Arsenie Gutan Department of Oral and Maxillofacial Surgery, Nicolae Testemitanu State University of Medicine and Pharmacy, 2004 Chisinau, Moldova; nicolae.chele@usmf.md (N.C.); andrei.mostovei@usmf.md (A.M.); gabriela.motelica@usmf.md (G.M.); ion.dabija@usmf.md (I.D.); stanislav.strisca@gmail.com (S.S.); mihaela.dandara@usmf.md (M.D.); liliana.nastas@usmf.md (L.N.); sergiu.beliniuc@usmf.md (S.B.); sofia.lehtman@usmf.md (S.L.); 8 Ion Lupan Department of Paediatric Stomatology, Nicolae Testemitanu State University of Medicine and Pharmacy, 2004 Chisinau, Moldova; elena.stepco@usmf.md; 9 Department of Physiology and Medical Biophysics, Nicolae Testemitanu State University of Medicine and Pharmacy, 2004 Chisinau, Moldova; svetlana.lozovanu@usmf.md; 10 Pavel Godoroja Department of Dental Propaedeutics, Nicolae Testemitanu State University of Medicine and Pharmacy, 2004 Chisinau, Moldova; olga.cheptanaru@usmf.md

**Keywords:** internal acoustic meatus, internal acoustic opening, morphometry, variability, facial nerve, vestibulocochlear nerve

## Abstract

**Background/Objectives**: The variability of the internal acoustic meatus (IAM) and its morphological peculiarities are of high clinical significance in otologic interventions and neurosurgery. Considering the complexity of the neurovascular structures housed by the IAM, including passage of the facial nerve, the aim of the study was to highlight the morphological peculiarities of the IAM and their clinical significance. **Methods**: The age peculiarities of the IAM course were studied on 18 serially, transversely sectioned human embryos and fetuses, as well as on 20 newborn and 20 adult formalin-fixed hemiheads. The morphometric parameters of the IAM (width, height, length, and angles between the anterior and posterior walls of the IAM and the longitudinal axis of the petrous ridge (LAPR) were evaluated on 82 dry temporal bones. **Results**: In embryos and early fetal stages of development, the course of the IAM formed an acute, posteromedially open angle with the longitudinal axis of the petrous part of the temporal bone (LAP); in newborns, it was a right angle; in adults, it was an acute, anteromedially open angle. Various shapes of the IAO, such as oval (51.2%), semioval (23.2%), quadrangular (13.4%), heart-like (3.7%), and triangular (8.5%), were highlighted. The mean width of the internal acoustic opening (IAO) was 7.7 ± 2.11 mm (right/left—8.2 ± 2.22 mm/7.2 ± 1.89 mm), with a statistically significant difference (*p* = 0.031). The mean height of the IAO was 5.3 ± 1.43 mm (right/left—5.6 ± 1.58 mm/5.0 ± 1.22 mm), *p* = 0.082. The mean length of the IAM was 10.1 ± 2.83 (right/left—10.5 ± 2.96 mm/9.7 ± 2.67 mm), *p* = 0.214. A partial septum was revealed in 3.6% of cases, while total septation of the IAM was observed in 2.4%. The mean angle between the anterior wall of the IAM and the longitudinal axis of the petrous ridge (AWIAM/LAPR) was 21.4 ± 13.67° (right/left—22.2 ± 14.62°/20.5 ± 12.78°), *p* = 0.564. The mean angle between the posterior wall of the IAM and the longitudinal axis of the petrous ridge (PWIAM/LAPR) was 82.4 ± 8.02° (right/left—84.3 ± 7.93°/80.5 ± 7.76°); *p* = 0.032. **Conclusions**: In the initial stages of an individual’s ontogenesis, the IAM forms an acute, posteromedially open angle with the LAP; in newborns—a right angle; in adults—an acute, anteromedially open angle. Both morphological characteristics and morphometric parameters of the IAO and IAM vary bilaterally. The oval, semioval, quadrangular, heart-like, and triangular shapes of the IAO were highlighted. Partial and total septation of the IAM were revealed. Only the TDAIO and PWIAM/LAPR angle were statistically significant, *p* < 0.05, while the bilateral correlation was negligible. The established morphological variants of the IAM could predispose to facial and vestibulocochlear nerve impairments.

## 1. Introduction

The internal acoustic meatus is an important anatomical structure that ensures passage, protection, and function of the vestibular ganglion, vestibulocochlear nerve, facial nerve, and labyrinthine vessels [1,2,3]. The common origin of the vestibulocochlear and facial nerves, from the facioacoustic primordium [4,5], and their vicinity within the internal acoustic meatus and at the bulbopontine angle, are predisposing factors for involvement of both nerves in various pathologies, including tumors.

Over the past few decades, the incidence of vestibular schwannomas has increased [6,7], with an intrameatal to extrameatal ratio of 1:3 [8], and an occurrence between 5 and 10% of all the intracranial tumors [6,9,10], accounting for around 80% of tumors of the cerebellopontine angle [10]. Historical rates of 0.7–1.0 per 100,000 population [6,11] have risen to 3–5 cases per 100,000 person-year, with rates reaching approximately 20 cases per 100,000 person-year among adults aged 70 and older [6,7]. The latest data reported an incidence of vestibular schwannoma exceeding 1 case among 500 people, while sporadic unilateral schwannomas account for about 95% of all cases, and about 90% of bilateral schwannomas develop in people with neurofibromatosis type 2 [6]. It should be noted that vestibular schwannoma can remain small and stable for a long time, or to grow rapidly with an unfavorable outcome [12]. In older patients, small schwannomas predominate, while in married [13] and young people [6], a larger size was reported. Along with tumor detection, computed tomography and magnetic resonance imaging allow identification of cochlear nerve hypoplasia, IAM stenosis, IAM duplication and malformations [14,15,16].

Advances in genomics, high-resolution imaging and functional studies have led to the identification of the major genes involved in anatomical and clinical variability of the IAM, vestibulocochlear and facial nerves. Their development is controlled by a complex of genes that coordinate otic placode induction, neurosensory differentiation, neural crest migration, and temporal bone osteogenesis.

The IAM develops by remodeling the petrous part of the temporal bone, and its development is influenced by both osteogenesis and the presence of the facial and vestibulocochlear nerves, which explains the association between cochlear nerve hypoplasia and stenosis of the IAM [14]. The genes RUNX2, FGFR2, TWIST1 and BMP4 are involved in osteogenesis and skull base remodeling [17,18,19,20]. Changes in these genes may influence the caliber and morphology of the IAM independently of the development of the vestibulocochlear nerve, explaining certain forms of stenosis or asymmetry of the IAM.

**Figure 1:**
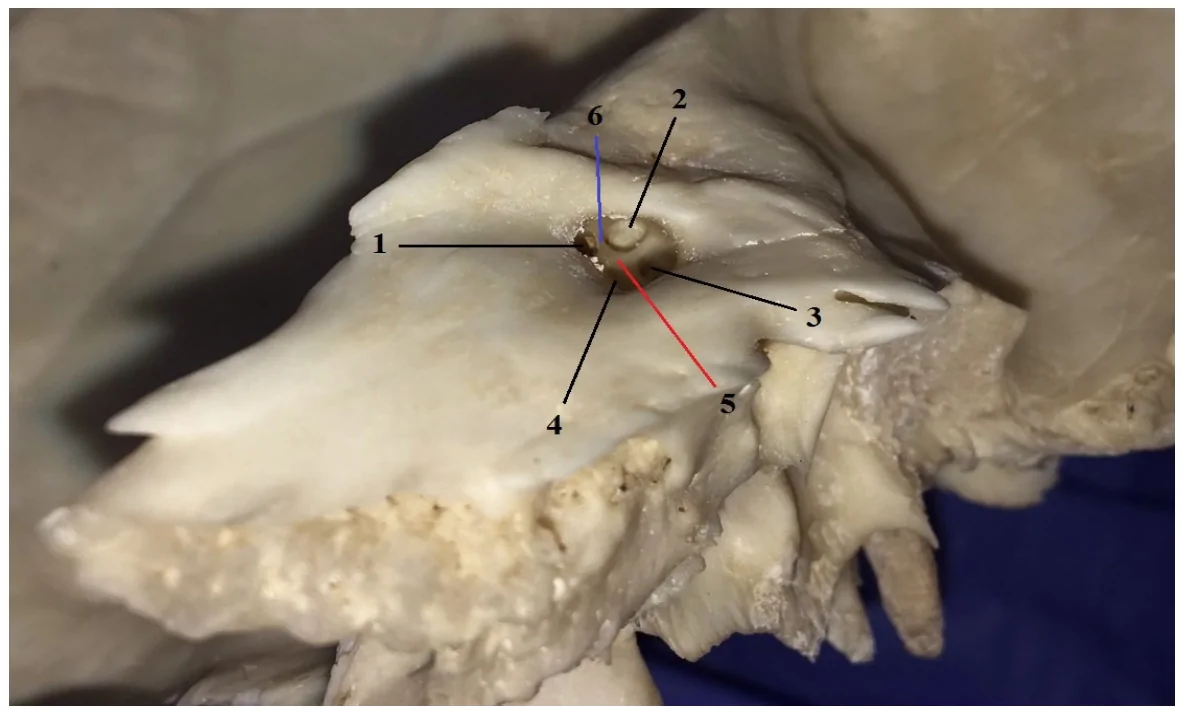
The bottom of the internal acoustic meatus of a right temporal bone: 1—anterosuperior area—inlet into the facial canal; 2—posterosuperior area or superior vestibular area; 3—posteroinferior area or inferior vestibular area; 4—anteroinferior or cochlear area; 5—crista falciformis or transverse crest; 6—Bill’s bar or vertical crest.

Anatomically, the internal acoustic meatus is a smooth, short, and rounded canal that starts on the posterior surface of the petrous part of the temporal bone at the internal acoustic opening and ends in a bony plate with cribriform areas, which separates it from the internal ear. It is approximately 10 mm in length and narrows towards its fundus. Its shape and size may be variable even in the same individual [21]. The bottom of the IAM is divided by the *crista falciformis* and Bill’s bar [16,22,23] into four areas (Figure 1).

In a clockwise way, the anterosuperior area transmits the facial nerve; through the posterosuperior area passes the superior branch of vestibular nerve, consisting of the utricular, anterior ampullary and lateral ampullary nerves; the posteroinferior area serves for passage of the inferior branch of vestibular nerve, formed by the saccular and the posterior ampullary nerves (the last nerve passing through the singular foramen); and the anteroinferior area transmits the cochlear nerve [16,22]. Along with the facial and vestibulocochlear nerves, the orifices transmit the arteries and veins of the organ of hearing and balance.

Abnormalities of the IAM and inner ear are identified in about 20% of patients with congenital sensorineural loss [14], and among those anomalies, in approximately 12% of cases, a narrow IAM was reported. Stenosis and other anatomical variations in the IAM can compromise both the nerves and labyrinthine vessels, resulting in nerve compression, tinnitus, and hearing loss [24,25]. The components of the IAM are highly susceptible to iatrogenic injuries in otologic surgery, particularly in vestibular schwannoma. The anatomical peculiarities, variants, and abnormalities of the IAM are of great clinical importance in terms of surgical access.

In the surgical approach from the middle cranial fossa, as a primary landmark, the greater petrosal nerve and its relationships with the neighboring anatomical structures is often used. Among the reliable landmarks were reported the angle between the IAM and longitudinal axis of the greater petrosal nerve [26]; the angles formed between the point of intersection of the greater petrosal nerve with the mandibular nerve and the base of the cochlea; the distance between the mandibular nerve at its origin from the trigeminal ganglion and fundus of the IAM [27]; the angle formed at the union of the foramen ovale with foramen spinosum and of the last one with the internal acoustic opening [28]. According to Rai et al. [29], the distances between the trigeminal ganglion and the medial surface of the posterior root of the zygoma; the lateral end of the petrous ridge; the arcuate eminence; and the hiatus of the greater petrosal nerve were statistically significant depending on laterality (*p* < 0.05).

One of the main problems in otologic surgery is preservation of the facial nerve function [10]. In various genetic syndromes associated with cochlear malformations, aberrant facial nerve pathways are at high risk of lesion, particularly in the context of cochlear implantation [14,30]. Due to multiple connections with the neighboring cranial and spinal nerves, the facial nerve might be involved in the pathology of those nerves [5,31,32], especially in impairments of the vestibulocochlear nerve. In order to avoid iatrogenic injuries to the facial nerve and labyrinthine artery, particularly in their variations [31,33], a cautious surgical approach is required.

Considering that after vestibular schwannoma surgery, the most common clinical symptom is sensorineural hearing loss [25], and about 30% of patients develop delayed facial nerve paralysis [34], the aim of the study was to highlight the anatomical specific features and morphological variations in the IAO and IAM, and their clinical significance.

## 2. Materials and Methods

The microscopic part of our research project was conducted in Minsk, on the basis of the Bilateral Agreement between the Department of Anatomy and Clinical Anatomy of Nicolae Testemitanu State University of Medicine and Pharmacy of the Republic of Moldova and the Department of Normal Anatomy of the Belarusian State Medical University from Minsk. The macroscopic observations and the morphometry were performed at the Department of Anatomy and Clinical Anatomy of Nicolae Testemitanu State University of Medicine and Pharmacy of the Republic of Moldova.

The age-specific features of the IAM were investigated in 18 groups of serially and transversely sectioned human embryos and fetuses from the historical embryo-fetal collection at the Department of Normal Anatomy of the Belarusian State Medical University. We studied 12 embryological groups, each including from 3 to 5 embryos (totaling 48 embryos), and six fetal groups, each including from 3 to 4 fetuses (totaling 22 fetuses).

The crown-rump length (CRL) of the embryos and fetuses varied from 9 mm to 57 mm. According to the available data, for fixation of the embryos and fetuses, a 10% formalin solution was used. The histological samples were stained using hematoxylin–eosin and Bielschowsky–Bucke silver impregnation methods. The morphometric and staining data of the samples were taken from the archived database of the Department of Normal Anatomy in Minsk. For protocol description, the OLYMPUS CX31 binocular microscope (SKU: OLY-CX31RBSFA/R-0326, Olympus Corporation, Ina Plant, Ina, Japan) (eyepiece 10×, objectives 4×; 10×; 40×; 100×) and Nikon DS-Fi1, DS-L2 (Nikon Corporation, Tokyo, Japan) were used.

Additionally, age peculiarities were examined on 20 newborn formalin-fixed hemiheads (10 right and 10 left) of the same individuals and on 20 adult formalin-fixed hemiheads (10 right and 10 left) of the same individuals from the Anatomy and Clinical Anatomy Department of Nicolae Testemitanu State University of Medicine and Pharmacy.

The morphometry was performed on 82 dry temporal bones (41 right and 41 left) from adult individuals, of unknown sex and age. Out of 82 dry temporal bones, 24 bones (12 right and 12 left) belonged to the same individuals, while 58 temporal bones (29 right and 29 left) belonged to different people. The transverse diameter of the internal acoustic opening (TDIAO); the vertical diameter of the internal acoustic opening (VDIAO); the length of the internal acoustic meatus (IAM length); the angle between the anterior wall of the internal acoustic meatus and the longitudinal axis of the petrous ridge (AWIAM/LAPR); the angle between the posterior wall of the internal acoustic meatus and the longitudinal axis of the petrous ridge (PWIAM/LAPR) were measured. The longitudinal and transverse diameters of the internal acoustic opening were measured with the Vernier caliper. The length of the internal acoustic meatus was measured using an anatomic needle, introducing it through the internal acoustic opening into the internal acoustic meatus until it reached the fundus. Upon extraction from the meatus, the needle was measured with the caliper. After measuring the length of the internal acoustic meatus, the anatomic needle was reintroduced into the IAM and fixed to its anterior wall. The angle between the needle and the longitudinal axis of the petrous ridge was measured with a protractor. Then, the needle was fixed to the posterior wall of the IAM, and the angle between the needle and the longitudinal axis of the petrous ridge was also measured. For intra-observer variability, the same observer took all the measurements twice, and for the final analysis, the average of the two measurements was used. The quantitative variables were analyzed using Excel “Descriptive statistics” option in “Tool—Data Analysis”, such as AVERAGE, STDEV, CONFIDENCE, CORREL, SKEW, etc. For the arithmetic mean of the quantitative variables, the confidence interval (CI_95_), with a safety level (*p*) of 0.95 and significance level (α) of 0.05, was calculated. To compare the variances between the two groups of measured parameters, the F-test was used. For comparison of the means of two independent samples, Student’s *t*-test was applied. The distribution of the variables and their symmetry were determined using the Excel SKEW function. For determining the bilateral correlation, the Pearson coefficient was used.

## 3. Results

**Figure 2:**
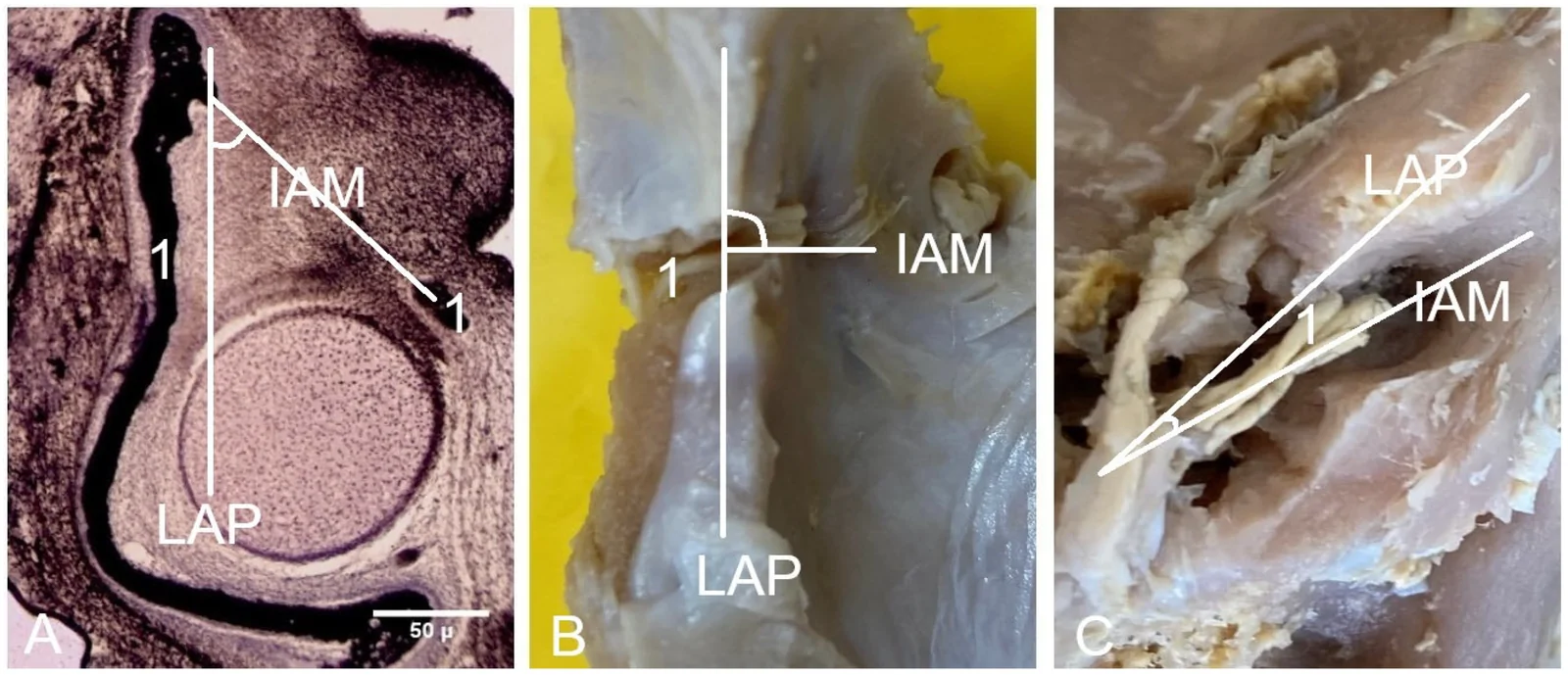
Angle between the internal acoustic meatus and longitudinal axis of the petrous part of the temporal bone. (**A**) Fetus with a CRL of 40 mm; (**B**) Newborn; (**C**) Adult; IAM—internal acoustic meatus; LAP—longitudinal axis of the petrous part of the temporal bone; 1—facial nerve.

The angle between the internal acoustic meatus and the longitudinal axis of the petrous part of the temporal bone changes during ontogenesis. The inlet into the internal auditory meatus and the facial nerve within it were first identifiable at Carnegie stage 20, by which point the angle between the IAM and the longitudinal axis of the petrous part of the temporal bone was already well defined. In embryos with a CRL of 20 mm and in fetuses with a CRL of 40 mm, that angle was acute and open posteromedially. In newborns, a right angle between the IAM and longitudinal axis of the petrous part of the temporal bone was identified, while in adults that angle was acute and directed anteromedially (Figure 2).

**Figure 3:**
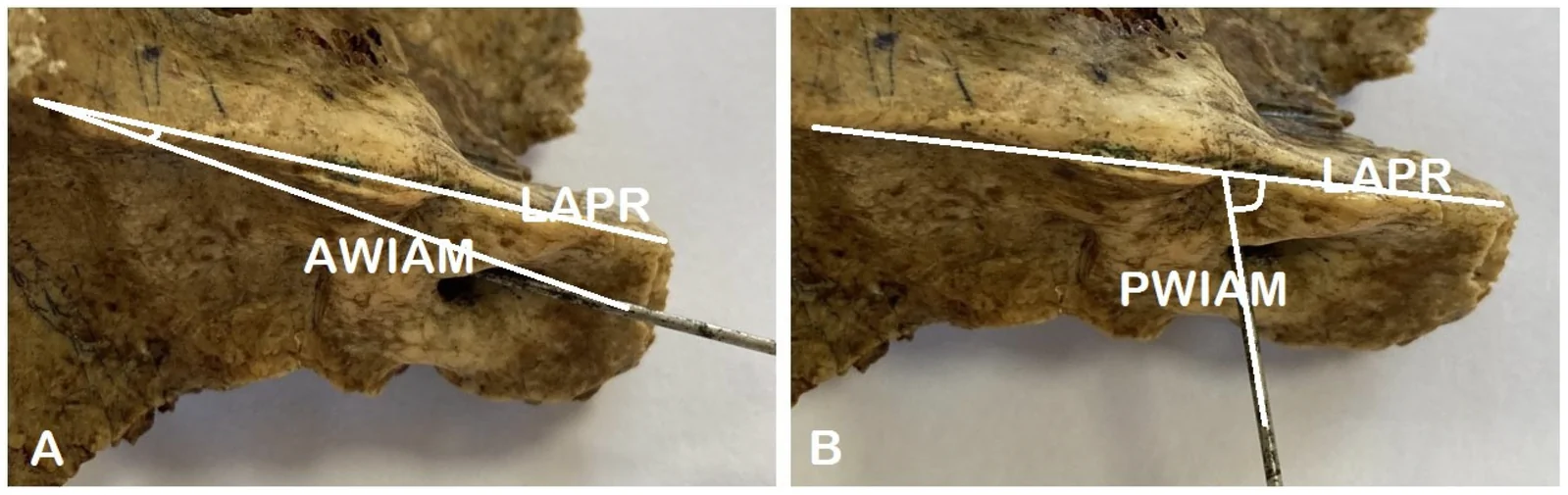
Angles between the anterior and posterior walls of the IAM and longitudinal axis of the petrous ridge. (**A**) Angle between the anterior wall of the internal acoustic meatus and the longitudinal axis of the petrous ridge (AWIAM/LAPR). (**B**) Angle between the posterior wall of the internal acoustic meatus and the longitudinal axis of the petrous ridge (PWIAM/LAPR).

A thorough examination of the temporal bones revealed a range of macroscopic specific features and variants of both internal acoustic opening and internal acoustic meatus. These included shape variants of the IAO, partial and total septation of the IAM, and variation in the angle between the anterior wall of the IAM and the longitudinal axis of the petrous ridge (AWIAM/LAPR). The angle between the posterior wall of the IAM and the longitudinal axis of the petrous ridge (PWIAM/LAPR) was also variable (Figure 3).

**Figure 4:**
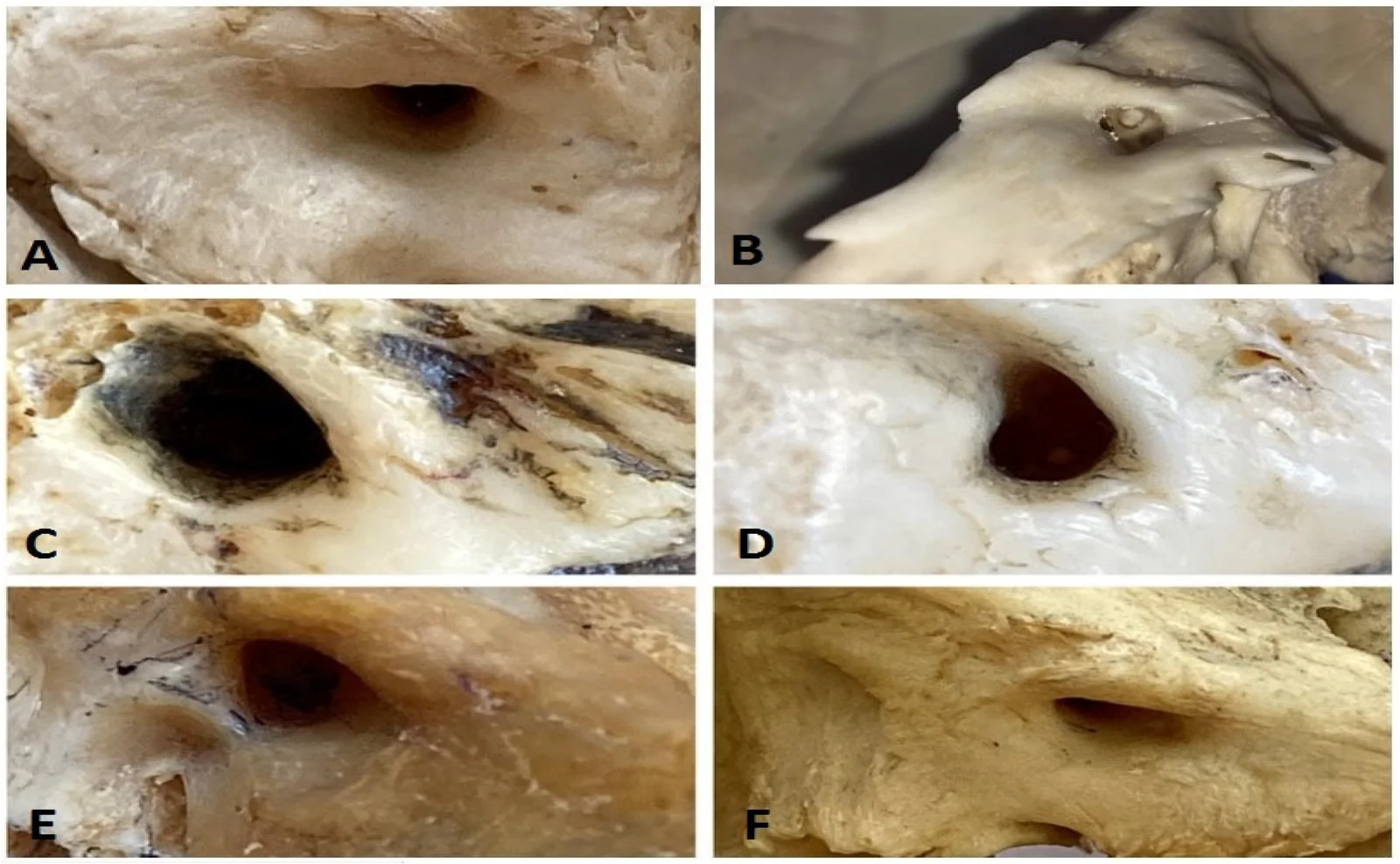
Variants of the internal acoustic opening. (**A**)—semioval; (**B**,**C**,**E**)—quadrangular; (**D**) heart-like; (**F**)—triangular (V-shaped).

A few shape variants were characteristic of the internal acoustic opening: oval, semioval, quadrangular, heart-like, and triangular (V-shape) (Figure 4). The oval shape was determined in 42 bones (51.2%), semioval—in 19 bones (23.2%); quadrangular shape—in 11 bones (13.4%); heart-like shape—in 3 bones (3.7%); triangular shape—in 7 bones (8.5%).

**Figure 5:**
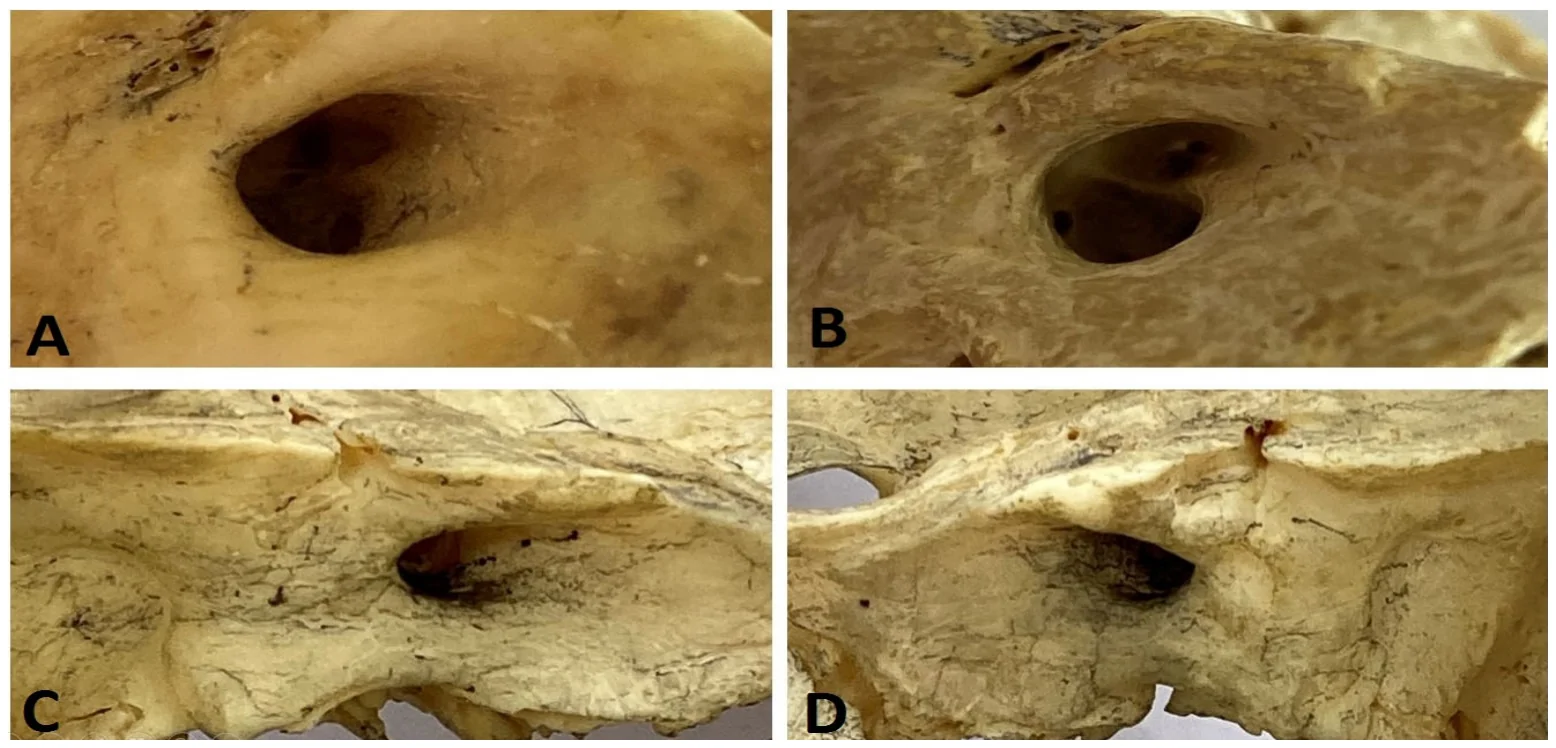
Septa of the internal acoustic meatus. (**A**,**B**) complete septum; (**C**,**D**) partial septum.

It should be noted that along with a straight and smooth course of the IAM, 3 cases of partial (3.7%) and 2 cases of complete septation (2.4%) were identified (Figure 5).

The mean transverse diameter of the internal acoustic opening (TDIAO) in the entire group was 7.7 ± 2.11 mm (max 12 mm–min 2 mm). For the right-side samples, the mean TDIAO was 8.2 ± 2.22 (max 12 mm–min 2 mm), and for the left-side samples, it was 7.2 ± 1.89 mm (max 12 mm–min 4 mm), with a statistically significant difference *p* = 0.031 (Table 1).

The mean vertical diameter of the internal acoustic opening (VDIAO) for the whole group was 5.3 ± 1.43 mm (max 9 mm–min 2 mm). For the right temporal bones, the mean VDIAO was 5.6 ± 1.58 (max 9 mm–min 2 mm), and for the left temporal bones, it was 5.0 ± 1.22 mm (max 8 mm–min 2.5 mm), *p* = 0.082 (Table 1).

The mean length of the IAM was 10.1 ± 2.83 (max 20 mm–min 6 mm) across all the examined temporal bones. On the right bones, the mean length of the IAM was 10.5 ± 2.96 (max 20 mm–min 6 mm), and on the left bones, it was 9.7 ± 2.67 mm (max 18 mm–min 6 mm), *p* = 0.214 (Table 1).

For the entire cohort, the mean angle between the anterior wall of the internal acoustic meatus and the longitudinal axis of the petrous ridge (AWIAM/LAPR) was 21.4 ± 13.67° (max 60–min 4°). When analyzed by side, the mean AWIAM/LAPR was 22.2 ± 14.62° (max 60–min 5°) on the right, and 20.5 ± 12.78° (max 52–min 4°) on the left, *p* = 0.564 (Table 1).

The mean angle between the posterior wall of the internal acoustic meatus and the longitudinal axis of the petrous ridge (PWIAM/LAPR) for the entire group was 82.4 ± 8.02° (max 93–min 59°). On the right side, the mean value was 84.3 ± 7.93 (max 93–min 59°), and on the left side, it was 80.5 ± 7.76° (max 90–min 62°), with a statistically significant difference depending on laterality, *p* = 0.032 (Table 1).

The Pearson correlation coefficient depending on laterality was very low for all the studied parameters: TDIAO (r = 0.003), IAM length (r = 0.312), PWIAM/LAP angle (r = 0.017), while for two of them the correlation was negative: VDIAO (r= −0.094) and AWIAM/LAPR angle (−0.171) (Table 1).

**Figure 6:**
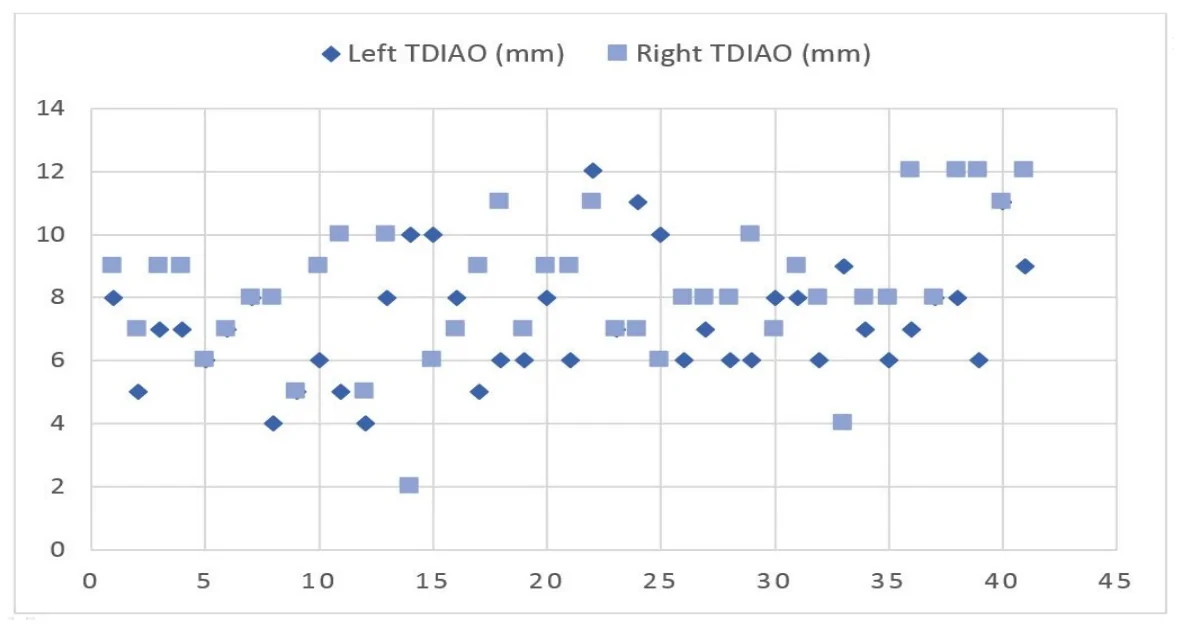
Correlation between the left TDIAO and right TDIAO.

**Figure 7:**
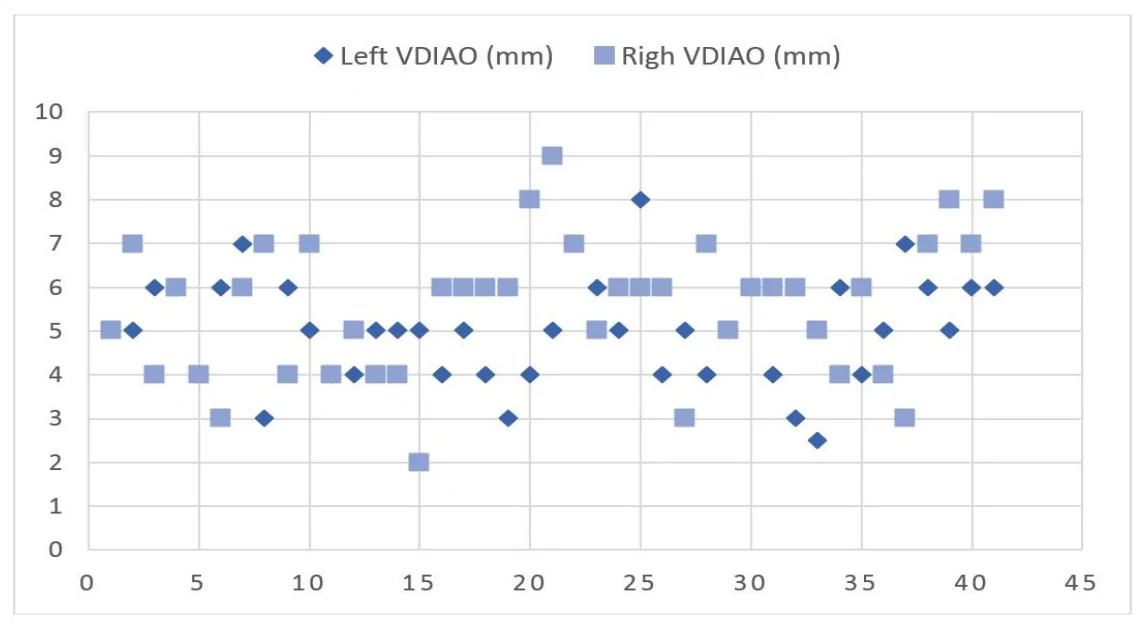
Correlation between the left VDIAO and right VDIAO.

**Figure 8:**
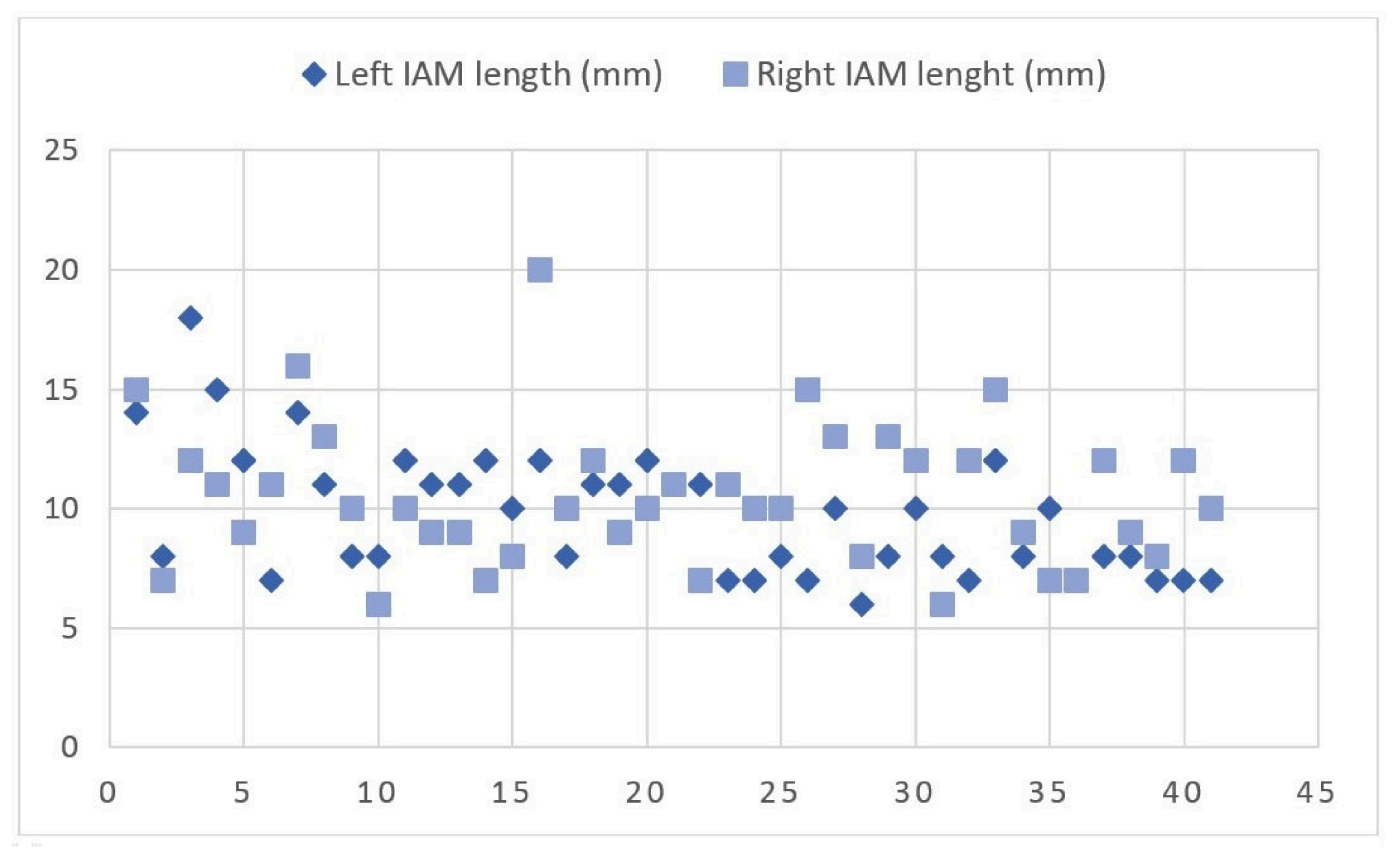
Correlation between the left IAM length and right IAM length.

**Figure 9:**
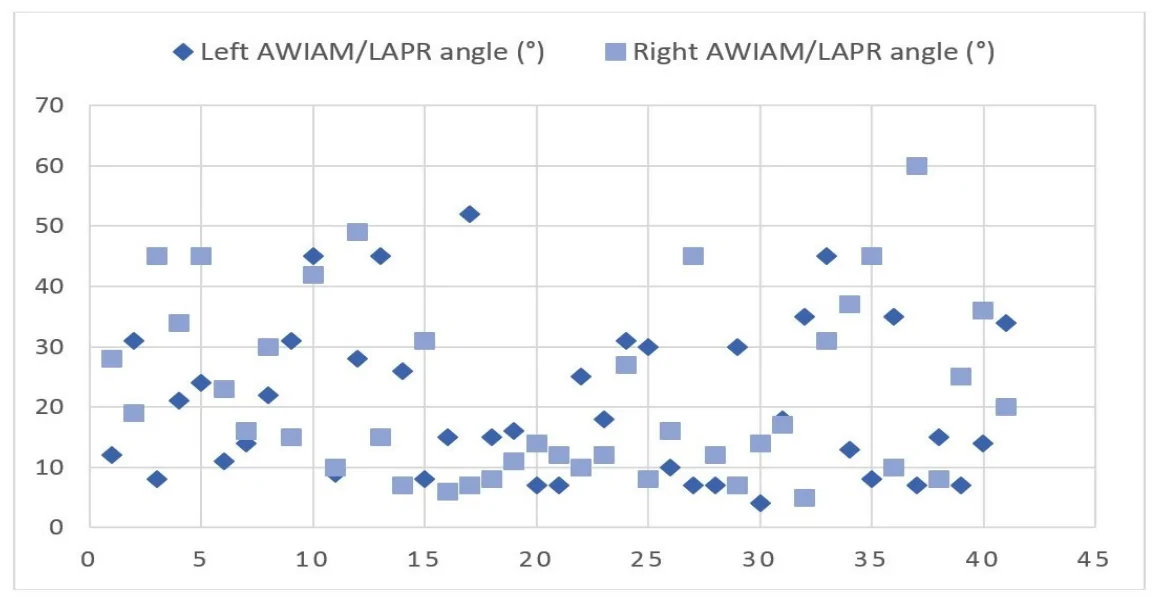
Correlation between the left AWIAM/LAPR and right AWIAM/LAPR.

**Figure 10:**
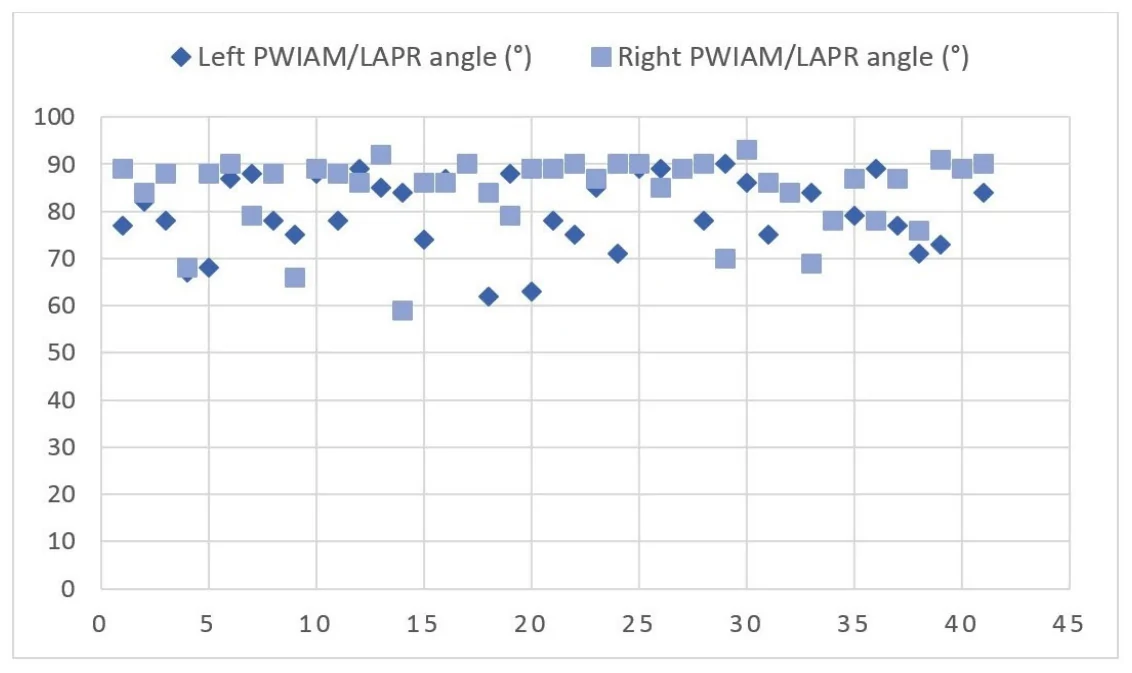
Correlation between the left PWIAM/LAPR and right PWIAM/LAPR.

Our data showed a statistically significant difference between the right and left TDAIO (*p* = 0.031), while a negligible association with laterality (r = 0.003) was revealed. The PWIAM/LAPR angle (*p* = 0.032) was also statistically significant, showing a weak association (r = 0.017) depending on laterality (Figure 6, Figure 7, Figure 8, Figure 9 and Figure 10).

## 4. Discussion

The internal acoustic opening and internal acoustic meatus are characterized by various shapes, presence of septa, stenosis, partial duplication, which occur due to disturbances of embryological development, or they can be acquired as a result of disrupted osteogenesis and some bone diseases [1,3,25,35].

The oval, irregular, fissure-shaped, U-shape, V-shape variants of the IAO [1,35] and elliptical IAO were reported [35]. In the current study, oval, semioval, quadrangular, heart-like, and triangular shapes of the IAO were identified.

The main morphological changes in the IAO occur until 10 years of age, preponderantly increasing its transverse diameter, while the vertical diameter continues its growth until 15–16 years of age. The IAO transverse diameter increases from 5 mm in a newborn up to 10 mm at ages of 15–17 years [35], while the IAM fundus attains adult dimensions at 21 weeks of intrauterine development [36]. The postnatal modifications of the IAM occur due to growth of the bone around the otic capsule [36], and the shape of the IAO and IAM could be influenced by the pneumatization of the temporal bone [35,36], as a result of which, the medial part of the IAM lengthens, and the medial edge of the IAO elevates [35]. The transverse diameter of the IAO is completed earlier than the vertical one [35].

According to Mamatha et al. [37], the mean height of the IAO on the right side was 3.56 mm, and the mean transverse diameter was 3.86 mm. On the left side, the vertical diameter had a mean of 3.44 mm, and the mean of the transverse diameter was 3.47 mm. Saygin et al. [1] reported a width of the right IAO of 6.83 ± 1.59 mm and of the left IAO of 6.31 ± 1.47 mm. The height of the right IAO was 4.12 ± 1.01 mm and of the left one, it was 4.54 ± 0.88 mm.

Marques et al. [15] obtained via CT examination a mean width of the IAO of 7.10 mm and a mean height of 4.47 mm, while Gözen et al. [38] reported a mean diameter of the right IAO of 7.91 ± 1.55 mm and of the left IAO of 8.10 ± 1.46 mm [38]. In the current study, the mean transverse diameter of the IAO was 7.7 ± 2.11 mm (right/left—8.2 ± 2.22 mm/7.2 ± 1.89 mm), and the mean vertical diameter was 5.3 ± 1.43 mm (right/left—5.6 ± 1.58 mm/5.0 ± 1.22 mm). Our morphometric parameters are consistent with those of Gözen et al. [38] and Marques et al. [15]. Nevertheless, Gözen et al. [38] reported a higher width of the left IAO, while we have obtained a higher width of the right IAO.

Some studies highlighted a slight predominance of the IAO width over its height [21,37], while in our study the ratio width/height (TDIAO/VDIAO) was 1.45:1. A range of authors reported asymmetric data of the IAM with prevalence of the left side parameters [21,38,39], while in our study the right parameters were higher. The asymmetry between the right and left sides in the current study could be conditioned by the heterogeneity of the temporal bones and a possible cranial asymmetry.

The most common normal variants of the IAM are the cylindrical and the funnel-shaped ones [15,40,41]. Mntungwa et al. [41] found the cylindrical shape in 57.14%, funnel shape in 40.48%, and bud shape in 2.38% of cases, while Marques et al. [15] reported a prevalence of funnel shape in both children and adults with a rate of 74%/55.3%, respectively, followed by cylindrical shape—22%/30.9% and the bud shape—4%/10.8%.

Özandaç et al. [21] obtained a mean length of the right IAM of 9.71 mm and a mean length of the left IAM of 9.92 mm. In our study, the IAM length was 10.1 ± 2.83 mm. Slightly higher values were obtained via CT by Gözen et al. [38], who determined on the right side a mean of 11.03 ± 1.63 mm and on the left side a mean of 11.13 ± 1.62 mm, and by Marques et al. [15], who obtained on CT a mean of the IAM length of 12.63 mm.

According to Özandaç et al. [21], in females, the length of the IAM on the right and left sides was 9.71 ± 1.46 mm and 9.92 ± 1.45 mm, respectively; in males, the length on the right and left sides was 9.61 ± 1.58 mm and 9.87 ± 1.64 mm, respectively. In our study, the mean length of the right IAM was 10.5 ± 2.96 mm and of the left IAM, it was 9.7 ± 2.67 mm. Our findings reveal a longer mean length of the right IAM compared with the left, a result that is inconsistent with the cited study.

In females, Özandaç et al. [21] reported a height of the right IAM of 4.65 ± 0.69 mm and of the left IAM of 4.66 ± 0.69 mm, while in males, the right and left mean values were 5.13 ± 0.85 mm and 5.13 ± 0.90 mm, respectively. In females, the IAM width on the right and left sides was 3.97 ± 0.6 mm and 3.95 ± 0.61 mm, respectively; in males, it was 4.18 ± 0.74 mm and 4.15 ± 0.71 mm, respectively.

Gibelli et al. [39] found that the left IAM was wider compared with the right one, and in females the lateral angle was higher than in males, correlating with widths of the IAM in both sexes. Even if the lateral angle in females was higher (45.5 ± 7.1) than in males (41.0 ± 6.7), and statistically significant (*p* < 0.01) [42], its cautious usage in sex differentiation was recommended [38,39,42].

The postnatal morphological changes mainly influence the width and the length of the IAM [43], while its volume gradually increases throughout an individual’s life, rising from 76.30 ± 24.24 (mm^3^) in early childhood (1 < 3 years), to 189.89 ± 61.75 (mm^3^) in adulthood (20–67 years) [2,36], significantly increasing until age of 10 years and without significant changes after late adolescence [36]. Conversely, the diameter of the IAM fundus remains constant across different age groups [43]. Maturation of the internal acoustic meatus is largely complete by 12–15 years of age [44]. Analyzing the data reported in the literature, we have noted that the morphometric parameters of the IAM vary among different ethnic groups, depending on sex and on age.

Variations in the configuration and size of the IAM are closely associated with the development of the vestibulocochlear nerve, which aplasia correlates with IAM abnormalities in 92% [45]. The hypoplasia or aplasia of the VIII pair of cranial nerves is frequently associated with congenital stenosis [46,47], agenesis [46], enlarged IAM, enlarged and narrowed fundus, defects of the lateral end of the IAM, hypoplasia or duplication of the IAM, suggesting a direct morphogenetic relationship between vestibulocochlear nerve and temporal bone development [14,46,48,49]. Stenosis of the IAM might be associated with the aberrant course of the facial nerve [47,50] and agenesis of the cisternal and intracanalicular parts of the vestibulochochlear nerve [47]. In cases of a dilated vestibule and malformation of the lateral semicircular canal, the facial nerve is displaced antero-medially, which increases its iatrogenic risk [50]. Along with shape and size variants, the IAM fundus is also variable. Double transverse crests, singular anterior crest splitting posteriorly into two branches, variable number of transverse crest foramina from one to four, an anterior crest structure narrowing the entrance into the facial canal, and a crest between foramina of the same nerve were highlighted [51]. In a rudimentary otocyst, the agenesis of the internal ear occurs, resulting in a common cavity communicating with the IAM [52], or an incomplete, extremely small oval or round otic capsule with agenesis of the IAM. The bulbous shape of the IAM and an incomplete separation of the lateral end of the IAM might result in communication of the subarachnoid space with the perilymphatic space of the cochlea [50,53,54], which can lead to recurrent meningitis [52,54].

Various landmarks were reported to be feasible in surgical access of vestibular schwannomas; nevertheless, the postoperative complications and mortality cannot be entirely avoided. Despite the possibility of facial nerve preservation, the use of the translabyrinthine approach in vestibular schwannomas results in total hearing loss [55,56,57], and surgeons often prefer the retrosigmoid approach with cerebellar retraction [57].

In anterior petrosectomy, for safe surgical access of the IAM as a reliable landmark, the angle formed between the lines connecting the foramen spinosum with the foramen ovale and with the internal acoustic meatus was reported. The mean value of that angle, on both dry skulls and human heads, measured radiologically, was 95 ± 1.97° [28]. An important peculiarity of the temporal bone is its pneumatization; the more extensive it is, the more laterally the labyrinth is located, facilitating the drilling of the IAM medially to the transverse crest [40].

Considering that the middle cranial fossa comprises anatomical structures highly susceptible to iatrogenic injuries and whose preservation is challenging in large vestibular schwannomas, the Kawase and extended Kawase approaches with a temporal lobe and Labbé’s vein retraction can be used to provide better exposure volume and area when the manipulation angle does not exceed 135° [58]. Gözen et al. [38] stated that an orientation angle around 180° provides a better translabyrinthine view during posterior cranial fossa surgical approach, while a lower angle would result in a narrower exposure. Even though the risk of iatrogenic injuries to the cavernous part of the internal carotid artery, oculomotor, trochlear, abducens, and facial nerves is still high [58]. Due to good exposure of the IAM, the facial nerve, and the geniculate ganglion, in tumors confined to the IAM, the middle cranial fossa access is the safest in terms of facial nerve protection, achieving a 60–80% rate in hearing preservation [59].

As feasible landmarks in the middle cranial fossa approach were reported, the distance between the point of the greater petrosal nerve intersection with the mandibular nerve and the base of the cochlea was measured, with a mean value of 10.46 ± 1.13 mm. Another reliable landmark is considered the distance between the origin of the mandibular nerve from the trigeminal ganglion and the fundus of the IAM, with a mean of 11.92 ± 1.71 mm [27]. Lesser et al. [30] established that the distance between the cochlea and adjacent anatomical structures, including IAM, does not differ between children older than 4 years and adults. However, in children younger than 4 years, those landmarks were significantly smaller.

According to Akansel et al. [42], the lateral angle of the IAM was higher in patients up to 17 years (45.8 ± 9.1°), with a non-significant decrease in patients older 18 years (42.9 ± 6.3°). Afacan et al. [60] found that the lateral angle is subjected to changes during postnatal ontogenesis. In children aged 0–2 years, the mean value of the lateral angle was 52.80 ± 9.95°, decreasing in children aged 5–12 years (46.49 ± 5.14°), and no significant changes were determined after 12 years of age.

A mean of 40.31 ± 8.01° was determined for the right lateral angle and a mean of 40.68 ± 8.92° for the left lateral angle by Gözen et al. [38], while the value of the orientation longitudinal axis of the IAM was 155.66 ± 11.19°. In our study, the mean values of both angles, AWIAM/LAPR (right/left—22.2 ± 14.62°/20.5 ± 12.78°) and PWIAM/LAPR (right/left—84.3 ± 7.93°/80.5 ± 7.76°), were higher on the right temporal bones, while the lateral angles evaluated by Gözen et al. [38] showed higher values on the left side. It should be noted that AWIAM/LAPR and the PWIAM/LAPR angles could not be used as essential surgical landmarks, while, along with the lateral angle, they can serve as additional landmarks to avoid the overextension of the neurovascular bundle when the neurinoma involves both the intracanalicular and the middle cranial fossa parts of the vestibulocochlear or facial nerves.

The current study highlighted important age-related peculiarities that characterize changes in the IAM course throughout an individual’s life. In embryonic and early fetal development, the course of the IAM formed a sharp posteromedially opened angle with the longitudinal axis of the petrous part of the temporal bone, while in newborns that angle was right and in adults it turned into a sharp anteromedially opened angle. To the best of our knowledge, there are no comprehensive studies about these peculiarities, and only Noren et al. [61] have noted that the angle between the facial and vestibulocochlear nerves and the brain stem with growth of the cephalic part of the embryo becomes sharper.

The otic capsule is a relatively stable structure, while the midline components of the skull elongate during intrauterine development. According to Hirano-Kawamoto et al. [62], at early embryonic stages the medial occipitopetrosal junction was located near the glossopharyngeal nerve, while at midterm of the intrauterine life, that junction was located in the immediate proximity of the internal acoustic meatus. The authors made a supposition that the growth of the skull might contribute to the sliding of the medial occipitopetrosal junction towards the spheno-occipital junction. During the first year of life, there is a rapid growth of the brain, which results in an increase in the neurocranium to about 65% of the adult skull, with predominant expenditure of the width of the petrous part of the temporal bone [63]. In fetuses at the age of 12 weeks, the developing tympanic cavity is almost horizontally positioned. In 19-week-old fetuses, a lateral movement of the temporal squama [64] and an increase in transverse diameter of the skull occur [65], resulting in lateral rotation of the inferior wall of the tympanic cavity and its shifting into a vertical position [64]. The petrous part of the temporal bone grows rapidly during the first year of life, both in width and in length [63,65], and the postnatal growth of the IAM occurs mainly due to growth of the bone tissue around the otic capsule [36].

In our opinion, those changes might contribute to rotation of the longitudinal axis of the IAM within the petrous part of the temporal bone, from the posterolateral to transverse and later, to anterolateral course. Considering that the orientation and position of the IAM can affect the visualization of the facial nerve in the translabyrinthine approach [44], our findings regarding ontogenetic morphological changes in the IAM course, towards the longitudinal axis of the petrous part of the temporal bone, are both of theoretical and clinical importance.

Some limitations of the current study should be noted, namely the low number of samples and the heterogeneity of the temporal bones, which were randomly collected from various people; the lack of sex and age information; the manual measurement of the investigated parameters; the modified edges of IAO on some temporal bones; and the evaluation of the parameters, based solely on laterality. Although the shape of the IAO was clearly distinguishable on the examined samples, the shape of the IAM was visible only in its initial part. This, combined with the inability to manually measure parameters within the IAM compared with CT imaging, represents a serious limitation of the current study.

## 5. Conclusions

An important ontogenetic peculiarity of the internal acoustic meatus course was highlighted in the current study. In embryonic and early fetal stages of development, the course of the IAM crossed the longitudinal axis of the petrous part of the temporal bone at an acute angle open posteromedially, while in newborns the IAM crossed the LAP at a right angle and in adults that angle became acute and open anteromedially. Both the internal acoustic opening and the internal acoustic meatus were bilaterally variable, and their mean morphometric parameters were higher on the right side. Five variants of the IAO shape were established: oval (51.2%), semioval (23.2%), quadrangular (13.4%), heart-like (3.7%), and triangular (8.5%). In 3.6% of cases, a partial septum and in 2.4% total septation of the IAM was observed. Only the TDAIO and PWIAM/LAPR angle were statistically significant, *p* < 0.05, while the bilateral correlation was negligible. The established morphological variants of the IAM could predispose to facial and vestibulocochlear nerve impairments.

## Figures and Tables

**Table 1:** The morphometric parameters of the internal acoustic opening and internal acoustic meatus.

Side and Number of Samples	Statistical Descriptors	TDIAO (mm)	VDIAO (mm)	IAM Length (mm)	AWIAM/ LAPR Angle (°)	PWIAM/ LAPR Angle (°)
Right side (41)	Mean value ± SD	8.2 ± 2.22	5.6 ± 1.58	10.5 ± 2.96	22.2 ± 14.62	84.3 ± 7.93
	Maximal value	12	9	20	60	93
	Minimal value	2	2	6	5	59
Left side (41)	Mean value ± SD	7.2 ± 1.89	5.0 ± 1.22	9.7 ± 2.67	20.5 ± 12.78	80.5 ± 7.76
	Maximal value	12	8	18	52	90
	Minimal value	4	2.5	6	4	62
Total (82)	Mean value ± SD	7.7 ± 2.11	5.3 ± 1.43	10.1 ± 2.83	21.4 ± 13.67	82.4 ± 8.02
	Maximal value	12	9	20	60	93
	Minimal value	2	2	6	4	59
	Difference	1.0	0.6	0.8	1.7	3.8
	*p*-value	0.031	0.082	0.214	0.564	0.032
	Standard error	0.23	0.16	0.31	1.51	0.89
	Dispersion	4.46	2.04	8.01	186.93	64.39
	Kurt Excess	−0.09	−0.28	1.05	−0.33	0.21
	Skew Asymmetry	0.13	0.10	0.91	0.79	−1.02
	Pearson coefficient	0.003	−0.094	0.312	−0.171	0.017
	Variation Coefficient (%)	27.29	27.04	27.96	63.99	9.74

SD—standard deviation, IAO—internal acoustic opening; IAM—internal acoustic meatus; TDIAO—transverse diameter of the internal acoustic opening; VDIAO—vertical diameter of the internal acoustic opening; IAM length—length of the internal acoustic meatus; AWIAM/LAPR angle—the angle between the anterior wall of the internal acoustic meatus and the longitudinal axis of the petrous ridge; PWIAM/LAPR angle—the angle between the posterior wall of the internal acoustic meatus and the longitudinal axis of the petrous ridge.

## Data Availability

The data reported in this study is available if required. The inquiry should be addressed to the corresponding author.

## Abbreviations

The following abbreviations are used in this manuscript:

IAM	Internal Acoustic Meatus
IAO	Internal Acoustic Opening
CRL	Crown-rump length
